# Near-resonance enhanced label-free stimulated Raman scattering microscopy with spatial resolution near 130 nm

**DOI:** 10.1038/s41377-018-0082-1

**Published:** 2018-10-24

**Authors:** Yali Bi, Chi Yang, Yage Chen, Shuai Yan, Guang Yang, Yaozu Wu, Guoping Zhang, Ping Wang

**Affiliations:** 1 Britton Chance Center for Biomedical Photonics, Wuhan National Laboratory for Optoelectronics-Huazhong University of Science and Technology, Wuhan, Hubei 430074 China; 2 MoE Key Laboratory for Biomedical Photonics, Collaborative Innovation Center for Biomedical Engineering, School of Engineering Sciences, Huazhong University of Science and Technology, Wuhan, Hubei 430074 China; 3 MoE Key Laboratory of Quark and Lepton Physics and College of Physics Science and Technology, Central China Normal University, Wuhan, 430079 China

## Abstract

High-resolution optical microscopes that can break 180 nm in spatial resolution set to conventional microscopies are much-needed tools. However, current optical microscopes have to rely on exogenous fluorescent labels to achieve high resolution in biological imaging. Herein, we report near-resonance enhanced label-free stimulated Raman scattering (SRS) microscopy with a lateral resolution near 130 nm, in which the high-resolution image contrast originates directly from a low concentration of endogenous biomolecules, with sensitivity gains of approximately 23 times. Moreover, by using a 0.3-m-long optical fiber, we developed hyperspectral SRS microscopy based on spectral focusing technology. Attributed to enhancements in spatial resolution and sensitivity, we demonstrated high-resolution imaging of three-dimensional structures in single cells and high-resolution mapping of large-scale intact mouse brain tissues in situ. By using enhanced high-resolution hyperspectral SRS, we chemically observed sphingomyelin distributed in the myelin sheath that insulates single axons. Our concept opens the door to biomedical imaging with ~130 nm resolution.

## Raman scattering microscopy: A better view for biology

Improvements in a technique called Stimulated Raman Scattering (SRS) allows high resolution biomedical imaging with optical microscopy, revealing biological structures without needing fluorescent chemical groups as ‘labels’. In SRS, laser light stimulates molecules into higher energy levels in which they scatter back light of different energy than the light stimulating them. Researchers in China, led by Ping Wang at Huazhong University of Science and Technology and Guoping Zhang at Central China Normal University, devised modifications that achieve resolution at an unprecedented level of around 130 nanometers. Careful selection and control of the laser light offers a 23 times gain in sensitivity over existing methods. Optical fiber technology allows the technique to be applied to intact tissues. Tests with a variety of cells and tissues demonstrate the potential for greatly enhanced biomedical imaging applications.

## Introduction

Label-free and high-resolution optical microscopes that can directly identify and image native biomolecules are highly desired^1–4^ but remain challenging. Advanced nonlinear imaging modalities, including pump–probe, four-wave mixing^5–7^, coherent anti-Stokes Raman scattering (CARS)^8–13^ and stimulated Raman scattering (SRS)^14,15^ microscopies, have been proposed in different approaches to improve spatial resolution, but only a few have been found to be very effective for biological systems.

For fluorescence imaging, it is straightforward to use nonlinear multiphoton microscopy for attaining an increase in imaging resolution of $\sqrt 2$ or more because the fluorescent signal is generated only at the very center of the focal spot of the laser^16^. However, the excitation laser wavelengths are strictly limited in the near-infrared (NIR) region because ultraviolet (UV) dyes or fluorescent proteins applicable for visible and nonlinear excitation are not readily available. In addition, visible femtosecond laser systems are not commercially available. Thus, the potential improvement in spatial resolution is completely compromised by the long wavelength adopted for nonlinear fluorescence imaging.

Fortunately, nonlinear CARS and SRS microscopies are free of limitations from labeling and applicable to this spot reduction effect. To fully utilize the nonlinear advantage to defeat the resolution limit, we reduced the wavelengths of our femtosecond lasers to the visible region^17^ and demonstrated visible SRS microscopy with subdiffraction resolution down to 130 nm. Meanwhile, the sensitivity of SRS increased by 23 times owing to near resonance and increased photon energy. Moreover, we adopted a 0.3-m-long polarization-maintaining single-mode (PM-SM) optical fiber to ensure excellent beam quality for high-resolution imaging and, importantly, achieved spectral focusing based hyperspectral SRS for selectively imaging biomolecules in intact tissues.

## Results

**Fig. 1:**
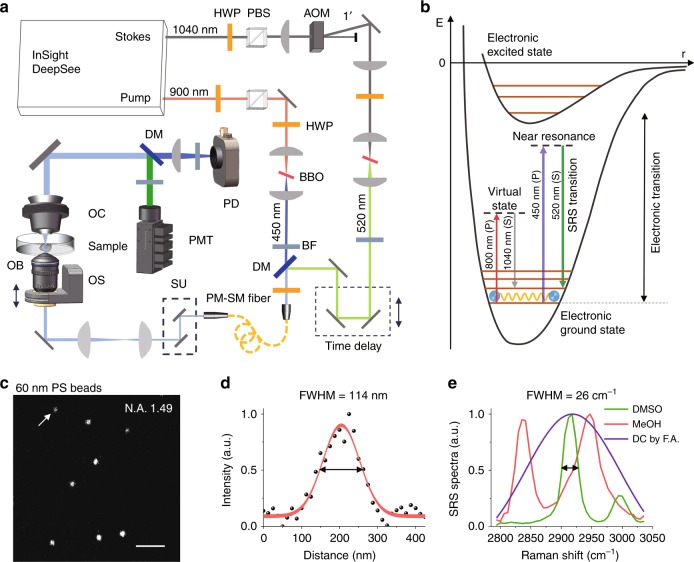
Visible SRS microscopy and spectroscopy. **a** Schematic of the visible SRS imaging system. HWP half wave plate, AOM acousto-optical modulator, BF bandpass filter, DM dichroic mirror, SU scanning unit, OS objective scanner, OB objective, OC oil condenser, PD photodiode, PMT photomultiplier tube, PM-SM polarization-maintaining single-mode fiber. **b** Energy diagram. **c** Visible SRS image of PS beads. **d** Cross-section profile of a selected PS bead in the boxed region in (**c**). The Gaussian fitting measured size of the bead is 114 nm (FWHM). **e** SRS spectra for MeOH and DMSO, which were normalized by the pump–probe signal of folic acid (FA) after Gaussian fitting. Scale bar, 1 µm

In the proof of concept of our high-resolution SRS microscope, the laser module outputs two femtosecond laser lines at wavelengths of 900 and 1040 nm (Fig. 1a, see setup details in the Materials and methods section). We effectively doubled the laser frequencies of our NIR femtosecond lasers by two beta-barium borate (BBO) crystals, with their wavelengths reduced in half to 450 and 520 nm, which served as pump and Stokes lasers, respectively. Figure 1b illustrates the energy diagram of our proposed concept. Since the nonlinear SRS signal is generated at the very center of the focal spot and complies with quadratic dependence of the excitation intensities, the spatial resolution naturally gains an additional $\sqrt 2$ in visible SRS imaging. Thus, the spatial resolution of this system determined by the Rayleigh criterion can be described as $d = \frac{{0.61\lambda _{\mathrm{em}}}}{{\sqrt 2 {\mathrm{N}}{\mathrm{.A}}{\mathrm{.}}}}$, where *λ*_em_ denotes the wavelength of the visible SRS excitation laser and N.A. is the numerical aperture of the applied objective. As we used a high-power oil immersion objective (N.A. 1.49) and short wavelength (*λ*_em_ = 450 nm) for excitation, the theoretical spatial resolution of our system can reach 130 nm. As shown in Fig. 1c, we performed visible SRS imaging for 60 nm polystyrene (PS) beads by detecting carbon–hydrogen (CH) vibrations at ~3050 cm^−1^ (Supplementary Fig. 3a); the cross-section profile of one bead is presented in Fig. 1d. The size of the full width at half maximum (FWHM) is ~114 nm by Gaussian fitting (see more experimental results in Supplementary Fig. 1e, f). After deconvolution of the actual size of the PS beads, we determined the point spread function (PSF) of our system to be ~107 nm. The spatial resolution defined by the Rayleigh criterion, which states the distance between two resolvable spots, is ~1.2 times the FWHM of the PSF^18–20^. In contrast to the high-resolution images obtained by the visible SRS microscope, the average FWHM of the PS beads imaged by NIR SRS was approximately 300 nm with a 60× water immersion objective (N.A. 1.2), and improved to 240 nm with a 100× oil immersion objective (N.A. 1.49, Supplementary Fig. 1a-d).

In addition to the improved spatial resolution, the increased photon energy also enhanced the SRS imaging sensitivity to endogenous biomolecules. In a conventional SRS microscope implementing NIR lasers to drive a coherent Raman transition, the intermediate virtual state is far detuned from the electronic excited state of the biomolecules. Here, we employed a visible laser to drive the near resonant stimulated Raman transition which, in theory, improves the coherent transition efficiency by approximately 50 times (see detailed calculation in the Materials and methods section). As a result, the laser powers required for further SRS imaging of biological samples are less than 5 mW for both the pump and Stokes beams.

To achieve molecular specificity along with improved imaging resolution and sensitivity, we implemented spectral focusing based hyperspectral SRS imaging in our concept^21,22^. Since the optical fiber shows a particularly high dispersion in the visible wavelength range, we adopted a 0.3-m-long PM-SM fiber to chirp the femtosecond laser pulses instead of applying high-dispersion glass rods. Importantly, the PM-SM fiber exhibits multiple advantages which ensures absolute collinearity for the pump and Stokes lasers, stabilized beam pointing and polarization, and an excellent Gaussian beam profile for high-resolution hyperspectral SRS imaging. To test and calibrate the proposed spectral focusing system, we measured the SRS spectra for dimethyl sulfoxide (DMSO) and methanol (MeOH) within a spectral window between 2800 and 3050 cm^−1^ (Fig. 1e). Considering that the laser energy has a certain distribution with wavelength, it is necessary to calibrate the SRS intensity in different Raman shifts^23^. After spectrum normalization by the transient absorption signal measured from folic acid (FA), the Raman bands of DMSO (2918 and 3000 cm^−1^) and MeOH (2835 and 2947 cm^−1^) were found to coincide with the spontaneous Raman spectra shown in Supplementary Fig. 3b.

**Fig. 2:**
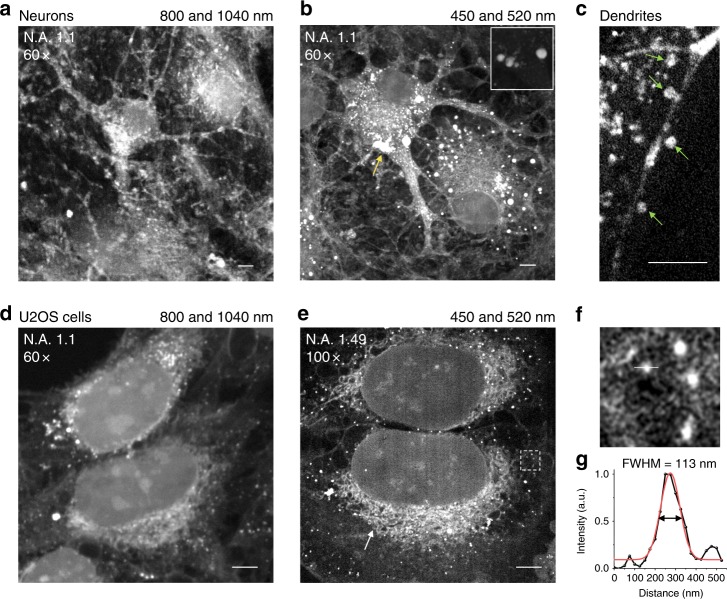
Visible SRS imaging of cultured neurons and U2OS cells. **a**, **b** Direct comparison of neuron images obtained by a conventional NIR SRS microscope (**a**) and visible SRS microscope (**b**). **c** Dendritic spines of the neuron. **d**, **e** NIR SRS image (**d**) and visible SRS image (**e**) of U2OS cells. **f** Zoom-in image of boxed area in (**e**). **g** Intensity profile of the indicated lipid droplet in U2OS cell in (**f**). The red curve shows the Gaussian fitting result for a lipid droplet size with a FWHM of 113 nm. Scale bars, 5 µm

After addressing challenges in improving spatial resolution, sensitivity, and molecular specificity, we demonstrated high-resolution SRS imaging of single cells. Figure 2a shows a vibrational image of cultured neurons acquired by a traditional NIR SRS microscope. As a direct comparison, the neurons imaged by the visible SRS microscope exhibit a significantly improved image contrast and spatial resolution, as shown in Fig. 2b. We observed the fine structures of the neurons which stretched out multiple dendrites with a clear boundary. Meanwhile, intracellular lipid droplets with different sizes were widely spotted in the soma and dendrites. It is worth noting that we also observed large lipid droplets in cultured neurons (pinpointed by a yellow arrow, inset in Fig. 2b) which were rarely found in the neurons in brain tissues. In Fig. 2c, we visualized the ultrafine spines distributed along the dendrites, as indicated by green arrows (microtubule-associated protein 2 (MAP2) immuno-labeled, see fluorescence and visible SRS images in Supplementary Fig. 4a, b). Interestingly, two glia cells were also observed by label-free SRS imaging, with each of them remaining close to one neuron. In addition, it is known that neurons are very active toward sensing and contacting with their neighbors. Strikingly, even for young neurons that were cultured in Petri dishes for only 7 days, densely distributed neural networks and connections could be clearly visualized (Supplementary Fig. 4c, d).

After installation of an oil immersion high N.A. (1.49, 100×) objective in the visible SRS system, we performed visible SRS imaging of U2OS cells (Fig. 2d, e). In comparison, the visible SRS image exhibited a substantially higher spatial resolution; we could visualize very fine mesh structures located outside of the cell nucleus which probably were endoplasmic reticulum (see more images in Supplementary Fig. 5). In Fig. 2f, we plotted the intensity profile across a lipid droplet, with the Gaussian fitted FWHM found to be 113 nm (Fig. 2g). By direct comparison, we conclude that the spatial resolution of the visible SRS microscope is significantly improved compared to traditional SRS.

**Fig. 3:**
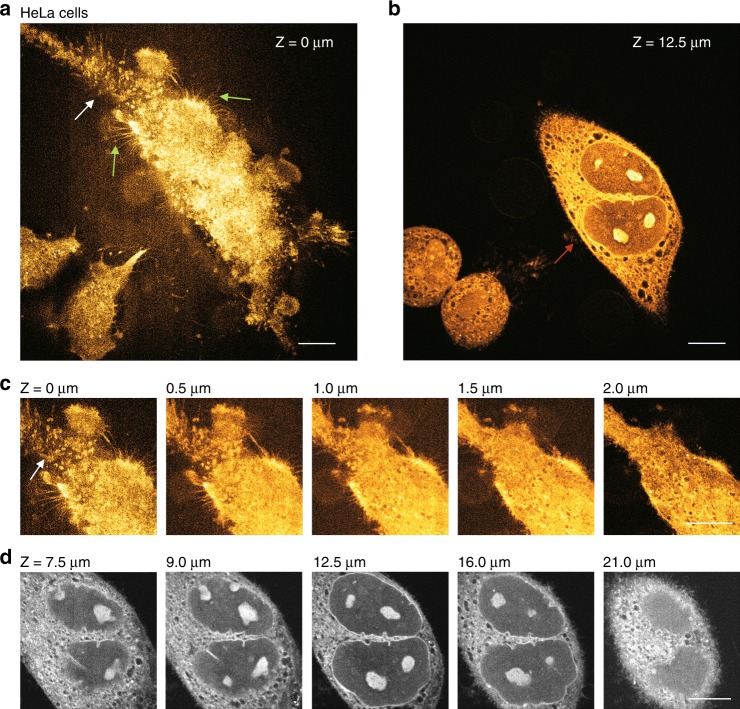
3D visible SRS imaging of cultured HeLa cells. **a**, **b** SRS images of cell body located mostly close to the planar glass surface (**a**) and at a height of *Z* = 12.5 μm (**b**). Water immersion objective with N.A. 1.1 was used for imaging. **c** Zoom-in images of the cell body at different heights corresponding to the location indicated by a white arrow in (**a**). **d** Images of cell nuclei at different heights corresponding to the location indicated by the red arrow in (**b**). Scale bars, 10 µm

A nonlinear SRS microscope naturally possesses the capability of optical sectioning; thus, we performed high-resolution three-dimensional (3D) SRS imaging of HeLa cells cultured in a Petri dish. In Fig. 3a, the cell body mostly located close to the planar glass surface (*Z* = 0 µm) was depicted in great detail. Actually, the cell body did not simply appear as a flat structure on the surface but rather was presented as a 3D structure. In the region indicated by the white arrow, we observed many irregular protrusions toward the surface of the glass which were probably responsible for cell migration as “feet”. In 3D stacked images (Fig. 3c), these tiny protrusions were extended out to approximately 1 µm in height from their cell body (see image at *Z* = 1.5 µm). In addition to these vertical protrusions, we also clearly visualized the delicate filopodia extending out horizontally from the basal membrane located on both sides of the cell body (indicated by green arrows) which may play essential roles in the sensing of surrounding environments. These cell filopodia are approximately 0.2 µm in diameter and 10 µm or less in length. Figure 3b shows a high-resolution SRS image of the nuclei of the same cell at *Z* = 12.5 µm. From the imaging planes scanned at higher positions between *Z* = 8 and 21 µm, we clearly observed 3D structures for both nuclear envelopes and the nucleolus with great detail (Fig. 3d). The complete 3D structures of the cells are presented in Supplementary Fig. 6 and Supplementary Video.

In fact, in contrast to NIR lasers, the visible lasers will cause severe photodamage to cells. In Supplementary Fig. 2, we characterized the level of photodamage to live HeLa cells as a function of total laser power and continuous laser scanning time. We found that the laser scanning time for safe live cell imaging decayed exponentially with increasing total laser power. When the total laser power was lower than 2.5 mW, the living cells could tolerate continuous laser scanning without a time limit. Thus, we have to balance the laser power and photodamage for extended live cell imaging. However, for fixed cells, we did not observe any apparent photodamage or thermal damage when the total laser power was below 10 mW.

**Fig. 4:**
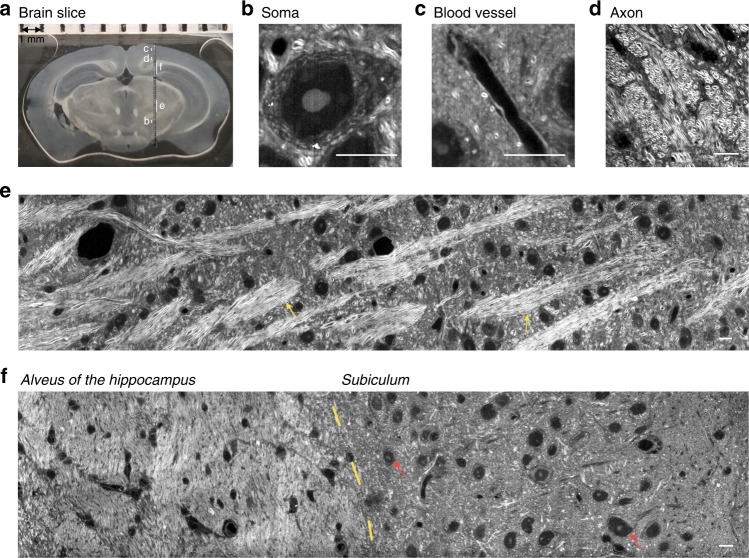
Visible SRS imaging of an unprocessed brain tissue section from a C57 wild-type mouse. **a** Overview of a coronal section of the brain slice. SRS inspected area is shown. **b**–**d** Enlarged views that illustrate the architectures of the soma (**b**), blood vessel in the cortex (**c**), and fiber bundles in white matter (**d**), with their locations marked by white lines (**b**–**d**) in (**a**), respectively. **e**, **f** High-resolution SRS imaging of brain areas corresponding to (**e**, **f**) indicated in (**a**), respectively. Scale bars, 10 µm

Due to difficulties in elimination of the out-of-focus fluorescence background, high-resolution imaging of intact tissue is challenging for fluorescence microscopes. To demonstrate the capability of direct tissue imaging, we demonstrated high-resolution SRS imaging of unprocessed brain tissue from a C57 mouse. As shown in Fig. 4a, a long strip area covering the cortex, *alveus of the hippocampus* (*alv*), *subiculum* (*S*)^24^, and other brain areas was examined (see detailed atlas of the mouse brain in Supplementary Fig. 7). In Fig. 4b, c, we present high-resolution SRS images of a neuron and part of one blood vessel in brain tissue (their relative locations are indicated in Fig. 4a). In the white matter of the brain, we observed a high density of myelinated axons (Fig. 4d). Since the myelin sheaths surrounding the axons are dominated by lipids, the SRS signal is much stronger than that from the other parts. Thus, in visible SRS imaging, the axons appeared as enclosed circles with a diameter of approximately 1 µm on the cross-section and two parallel curves on the longitudinal section. As shown in Fig. 4e, the neural networks were weaved of crisscrossed fiber bundles (indicated by yellow arrows), among which blood vessels and neurons were vastly distributed. Especially in the *alv* region, fiber bundles of axons were packed with unprecedented density in all directions (Fig. 4f). We also observed a clear boundary that divided *alv* and *S* regions, where the distribution density of the somas and axons exhibited great differences. In the *S* region, we found densely populated neurons (indicated by red arrows), but much fewer fiber bundles. The high-resolution SRS maps covering the complete inspected area of the brain tissue (indicated in Fig. 4a) are exhibited in Supplementary Fig. 9. To evaluate the imaging depth for visible SRS imaging, we performed 3D imaging of white matter in a tissue slice of mouse brain. As shown in Supplementary Fig. 8, we directly compared the imaging depth of our system with that of an NIR SRS system. We found that the visible SRS imaging depth was approximately 10 µm with decent image contrast. For the NIR SRS microscope, the penetration depth is approximately 50 µm in a similar region.

**Fig. 5:**
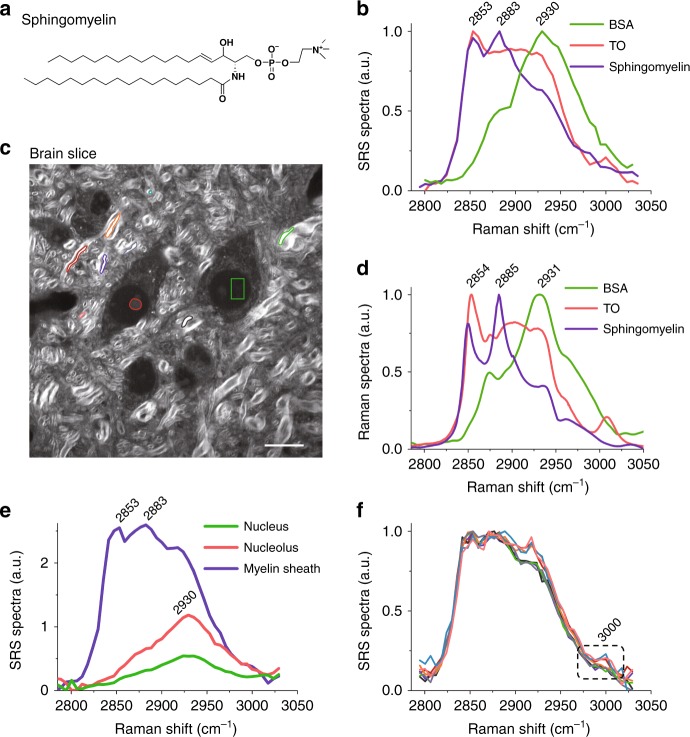
Hyperspectral visible SRS imaging of mouse brain tissue. **a** Chemical structure of sphingomyelin. **b** SRS spectra for BSA, TO, and sphingomyelin in the Raman region of 2795–3035 cm^−1^. **c** Hyperspectral visible SRS image stack of a brain slice. Single SRS image at a Raman shift of 2,920 cm^−1^ is shown. **d** Spontaneous Raman spectra corresponding to (**b**). **e** SRS spectra for a myelin sheath, nucleus, and nucleolus acquired at the locations indicated in (**c**). **f** SRS spectra for myelin sheaths at different locations circled in (**c**). Scale bar, 10 µm

Based on significantly improved spatial resolution, we further performed hyperspectral SRS imaging of mouse brain tissue^25^. It is known that the major chemical compositions of brain tissues are sphingomyelin, neutral lipids, and protein. As shown in Fig. 5a, sphingomyelin comprises two straight long acyl chains in its chemical structure. To differentiate the major chemical compounds in mouse brain tissue, we acquired SRS spectra for pure chemicals including sphingomyelin, glyceryl trioleate (TO), and bovine serum albumin (BSA, a protein representative), as shown in Fig. 5b. Except for the symmetric CH_2_ vibrational band at 2853 cm^−1^^26^, TO exhibits a distinctive Raman feature for lipid unsaturation at 3005 cm^−1^ which arises from vibrational stretching of =CH^27–29^. In contrast to unsaturated TO, sphingomyelin presents a characteristic Raman band at 2883 cm^−1^ which is the signature band for saturation that possibly originated from Fermi resonance or asymmetric vibration of (CH_2_)_n_ in the long acyl chain^30,31^. In addition, protein presents a prominent Raman band at 2930 cm^−1^ due to strong vibration of symmetric CH_3_. To verify the SRS spectra, Fig. 5d illustrates the corresponding spontaneous Raman spectra which are consistent with the major features found in the SRS spectra.

We further examined unprocessed mouse brain tissue by a hyperspectral visible SRS microscope. A stack of 45 SRS images (800 × 800 pixels per image) was obtained at wavenumbers ranging from 2795 to 3035 cm^−1^. Thus, we were able to record the Raman spectra and analyze the composition of the tissue at each pixel of the high-resolution image (SRS image at 2920 cm^−1^ is presented in Fig. 5c). Figure 5e shows the SRS spectra collected at pinpointed locations in Fig. 5c, where a myelin sheath of axons (circled in purple), neuronal nucleus (green) and nucleolus (red) can be seen. As previously discussed, the lipid droplets largely distributed in the cultured neurons were seldom found in brain tissue. Meanwhile, the Raman spectra for the nucleus and nucleolus presented a strong peak at 2930 cm^−1^, suggesting that their dominant contents are proteins. As expected, we specifically observed the signature Raman band of sphingomyelin at 2883 cm^−1^ at the locations of all the axons (Fig. 5e, blue), which implies that the insulating layers of myelinated axons are densely wrapped up with saturated fat, sphingomyelin. In further investigations, we also found that the myelin sheath contained unsaturated fat along with saturated fat at some locations, as shown in Fig. 5f, where the spectra show an emergent Raman band of 3005 cm^−1^. This band is possibly due to the existence of some amount of phosphatidylethanolamine, phosphatidylserine, or other unsaturated fats in a typical myelin sheath^32^. However, further verification regarding the chemical contents of the myelin sheath is required.

## Discussion

In summary, we developed label-free visible SRS microscopy with a spatial resolution near 130 nm and demonstrated high-resolution imaging of low-concentration endogenous biomolecules in cells and tissues with great sensitivity. Moreover, optical fiber-based spectral focusing technology enabled high-resolution biochemical analysis for intact tissues. For further research, most potential applications of visible SRS microscopy can be extended to imaging widely available visible dyes, other than NIR dyes, for supermultiplexing microscopy.

## Materials and methods

### Visible stimulated Raman scattering microscopy

We employed a dual-output femtosecond laser system (InSight DeepSee, Spectra-Physics, Newport) to provide two phase-locked femtosecond lasers with a repetition rate of 80 MHz. The 220 fs output laser at a wavelength of 1040 nm was modulated by an acousto-optical modulator (AOM, 1205C-1, Isomet) at 2.185 MHz with 100% modulation (the first diffraction order of AOM was adopted). To generate short laser wavelengths for visible SRS imaging, we implemented a BBO crystal (4 × 4 × 0.8 mm, SHG@1040 nm, Union Optic) to double the frequency of the 1040 nm laser to 520 nm, which served as a Stokes beam. Similarly, the second 100 fs laser at 900 nm was frequency doubled by the second BBO crystal (4 × 4 × 0.6 mm, SHG@750-1100 nm, Castech) to a 450 nm laser, which served as a pump beam. A time delay device based on a motorized stage (TLS28EKT04U/TDC15, Zaber) was applied to obtain SRS spectra in spectral focusing. After spatially overlapping the pump and Stokes lasers through a dichroic mirror (ZT491dcsp, Chroma Technology), both lasers were coupled into a 0.3-m-long optical fiber (LPC-04-450-3/125-P-1.6-10AC-40-3AF-3-0.3, OZ Optics) to chirp the pulse width for spectral focusing. The two color collinear Gaussian beams were guided into a custom-built inverted microscope equipped with a two-axis galvanometer (GVS002, Thorlabs). For high-resolution imaging, we used three high N.A. objectives (N.A. 1.2, UPLSAPO 60XW; N.A. 1.1, LUMFLN 60XW; N.A. 1.49, UAPON 100XOTIRF, Olympus) in various experiments. An objective scanner (P-725.4CD, Physik Instrumente) was installed for 3D cell imaging. After interaction with samples, the transmission lasers were collected by a high N.A. oil condenser (N.A. 1.4, U-AAC, Olympus) and detected by a Si PIN photodiode (S3994-01, Hamamatsu), which was equipped with a 2.1 MHz resonant amplifier. To block the modulated 520 nm laser, two high-quality bandpass filters (ET450/40M/2P, Chroma Technology) were installed in front of the detector. To demodulate the visible SRS signal, we implemented a high-speed lock-in amplifier (HF2 LI, Zurich Instruments) for sensitive heterodyne detection. For fluorescence imaging, a 450 nm pump laser was applied as excitation, with two bandpass filters (ET525/50m-2p, ET525/70m-2p, Chroma Technology) placed before a photomultiplier tube (H7422-40, Hamamatsu) used to block all scattering photons except for fluorescence. All imaging signals were acquired by a 1.25 MHz acquisition card (PCI 6251 NI, National Instrument).

### Laser settings and data acquisition

The laser powers were measured before the objective for all experiments. For PS bead imaging in Supplementary Fig. 1a, c, the pump and Stokes lasers were set at 792 nm (30 mW) and 1040 nm (45–55 mW), respectively. As shown in Supplementary Fig. 1a, the dwell time for NIR SRS imaging was 10 μs/pixel, with a field of view of 80 × 80 μm^2^ with 2000 × 2000 pixels. As shown in Supplementary Fig. 1c, the field of view was 40 × 40 μm^2^ with 1600 × 1600 pixels, and the dwell time for imaging was 50 μs/pixel. For PS bead imaging in the visible SRS system (Fig. 1c, Supplementary Fig. 1e), we applied an oil immersion objective (100×, N.A. 1.49). The pump and Stokes lasers were set at 449.5 nm (1.7 mW) and 520 nm (1.6 mW), respectively. The field of view was 20 × 20 μm^2^ with 1600 × 1600 pixels, and the dwell time was 10 μs/pixel. For visible SRS imaging of biological samples, the laser powers were 3–5 mW for both the pump and Stokes beams. The dwell times and pixel settings were 20 μs/pixel, 2000 × 2000 pixels for U2OS cells (Fig. 2d, e, Supplementary Fig. 5); 40 μs/pixel, 1600 × 1600 pixels for neurons (Fig. 2a, b, Supplementary Fig. 4c, d); 30 μs/pixel, 1600 × 1600 pixels for HeLa cells (Fig. 3, Supplementary Fig. 6, Supplementary Video); and 35 μs/pixel, 2000 × 2000 pixels for brain tissue (Figs. 4, 5, Supplementary Figs. 7, 9 and 8d-f). For the fluorescence imaging in Supplementary Fig. 4a, b, the pixel setting is 800 × 800 pixels with a dwell time of 10 μs/pixel. For the cellular viability test in Supplementary Fig. 2, we used dwell times of 10 μs/pixel, with a field of view of 100 × 100 μm^2^ with 800 × 800 pixels per frame. For the NIR SRS imaging in Supplementary Fig. 8a-c, the power of both pump and Stokes beams was 40 mW, and the pixel setting was 400 × 400 pixels with dwell time of 10 μs/pixel. For hyperspectral SRS imaging of brain tissue (Fig. 5), we applied a water immersion objective with N.A. 1.2, with pump and Stokes beam laser powers of 4.2 and 2.6 mW, respectively.

### Sensitivity analysis for visible SRS imaging

When the energy difference between the pump and Stokes photons matches the vibrational energy of the target molecules, the coherently induced vibrational transition absorbs one photon (stimulated Raman loss (SRL)) in the pump beam and gains one photon in the Stokes beam (stimulated Raman gain (SRG)). The signal for SRL can be expressed by

1 $$\Delta I_{\mathrm {p}} \propto - N \times \sigma _{\mathrm {Raman}} \times I_{\mathrm {p}} \times I_{\mathrm {S}}$$

Here, Δ*I*_p_ denotes the relative loss in the pump laser, *N* is the number of vibrational bonds in the imaging volume, *σ*_Raman_ is the Raman scattering cross-section, and *I*_p_, *I*_S_ are the laser intensities of the pump and Stokes beam, respectively^15^.

Importantly, *σ*_Raman_ can be significantly increased by near resonant condition. *σ*_Raman_ can be described as^33–35^

2 $$\sigma _{\mathrm {Raman}} = K\omega _{\mathrm {pump}}\omega _{\mathrm {Stokes}}^3\left[ {\frac{{\omega _{\mathrm {pump}}^2 + \omega _e^2}}{{\left( {\omega _{\mathrm {e}}^2 - \omega _{\mathrm {pump}}^2} \right)^2}}} \right]^2$$

Here, *ω*_pump_ and *ω*_Stokes_ are the pump and Stokes laser frequencies, respectively; *ω*_e_ is the frequency of the laser that can drive an electronic transition, and *K* is a constant related to the target biomolecules. Obviously, for *ω*_pump_ « *ω*_e_, *σ*_Raman_ will increase by 16 times after frequency doubling. In typical UV absorption spectra for biomolecules, lipids or fatty acids are responsible for absorption at ~150 nm due to the σ → σ^*^ transition of the carbon–hydrogen vibrations. DNA and proteins show different but strong absorption bands at ~260 and 280 nm^36^, which are due to the *n* → *π* ^*^ transition of the aromatic groups.

According to formula (2), *σ*_Raman_ actually increases by 23 times, as the energy of the electronic transition corresponds to 150 nm. Meanwhile, the intensity of the pump and Stokes lasers (*I*_p_, *I*_S_) can increase 4 times each because of the smaller focal spot. Moreover, the molecule number *N* will decrease 8 times due to reduced focal volume. Thus, the SRS intensity (Δ*I*_p_) of visible SRS is approximately 46 times more than that of an NIR laser-based SRS. Considering that the shot noise proportionally increases with photon energy, the factor of the sensitivity enhancement should be 23.

### Sample preparation for visible SRS and fluorescence imaging

#### Pure samples

Droplets of DMSO (30072418, Sinopharm Chemical Reagent Co., Ltd), MeOH (10014118, Sinopharm Chemical Reagent Co., Ltd), FA (dissolved in double steamed water, F7876, Sigma-Aldrich), TO (T7140, Sigma-Aldrich), powder of BSA (4240GR005, BioFroxx), and sphingomyelin (S0756, Sigma-Aldrich) were sealed between two cover glasses (48393-172, VWR) for different experiments.

#### Visible SRS imaging of PS beads

PS beads (0.06 μm mean particle size, Spherotech, Inc.) were resuspended by vortexing with deionized water diluted to an appropriate concentration. Then, the solution of PS beads was sonicated for 20 min at room temperature to obtain a homogeneous solution, followed by a 1:1000 dilution with deionized water^37^. Before visible SRS imaging, the beads were sealed between two glasses. We dried the beads on one cover glass and immersed them in pure oil (glyceryl trioleate, TO) to avoid Brownian motion.

#### Cell lines

HeLa cells (Fig. 3, Supplementary Figs. 2, 6, Supplementary Video) were cultured in Dulbecco’s modified Eagle's medium (DMEM, Thermo Fisher Scientific, Inc.); the human osteosarcoma cell line (U2OS, Fig. 2d, e, Supplementary Fig. 5) cells were cultured in McCoy’s 5A medium (Thermo Fisher Scientific, Inc.). All media were supplemented with 10% fetal bovine serum (Thermo Fisher Scientific, Inc.) and 1% penicillin–streptomycin (BasalMedia) and maintained in a humidified atmosphere with 5% CO_2_ at 37 °C^38^.

#### Visible SRS imaging of fixed HeLa and U2OS cells

HeLa and U2OS cells were seeded in Petri dishes (glass bottom Φ 20 mm, Cellvis) at a density of 1 × 10^5^ per well with 1 ml DMEM/McCoy’s 5A medium for 24 h and then fixed for 10 min at room temperature with 4% paraformaldehyde (PFA)^39^. After fixation, cells were washed three times with phosphate-buffered saline (PBS, pH of 7.4, Gibco) before imaging.

#### Mice and tissues

We have complied with all relevant ethical regulations, and all animal experiments were approved by the Institutional Animal Ethics Committee of Huazhong University of Science and Technology. The brain tissue from a 4-week-old C57BL/6J female mouse (Figs. 4, 5, Supplementary Figs. 7, 9 and 8d-f) was sliced and washed once by PBS before sealing between two coverslips. The thickness of the tissue slice is 50 μm in Fig. 4 and 70 μm in Fig. 5.

#### Immunofluorescence staining of fixed neurons

Neurons (Supplementary Fig. 4a, b) cultured on glass coverslips in 24-well plates were fixed with 4% PFA for 10 min, and then washed with PBS three times. After a permeabilization process with 0.2% Triton X-100 for 10 min, all neurons were treated with a blocking solution (3% BSA in PBS) for 30 min and rinsed twice with PBS^40^. The primary antibody, anti-MAP2 antibody in rabbit (ab32454, Abcam), was diluted by 1:1000 in a 3% BSA solution and added into plates overnight at 4 °C. Then, the neurons were rinsed three times with PBS. A goat-anti-rabbit Alexa488 (ab150077, Abcam) secondary antibody was applied followed by a 1:500 dilution in 3% BSA solution, and cells were incubated for 1 h at room temperature in the dark^33^. These glass coverslips were moved to Petri dishes (glass bottom Φ 20 mm, Cellvis) filled with PBS. Neurons were washed three times with PBS before imaging.

## Electronic supplementary material

Supplementary Fig. 1-8

Supplementary Fig.9

Image stack of the HeLa cells
