# Supplementary material for: Near-resonance enhanced label-free stimulated Raman scattering microscopy with spatial resolution near 130 nm
Source: Light Sci Appl. 2018 Oct 24;7:81. doi: 10.1038/s41377-018-0082-1 (PMC6199294; doi:10.1038/s41377-018-0082-1)
Supplement: Supplementary file 1 — Supplementary Fig. 1-8 [file 41377_2018_82_MOESM1_ESM.pdf]

Near-resonance Enhanced Label-free Stimulated Raman Scattering Microscopy with Spatial Resolution near 130 nm

Yali Bi[1, 2, 3#], Chi Yang[1, 2#], Yage Chen[1, 2], Shuai Yan[1, 2], Guang Yang[1, 2], Yaozu Wu[1], Guoping Zhang[3*] & Ping Wang[1, 2*]

[1]Britton Chance Center for Biomedical Photonics, Wuhan National Laboratory for Optoelectronics-Huazhong University of Science and Technology, Wuhan, Hubei 430074, China

[2]MoE Key Laboratory for Biomedical Photonics, Collaborative Innovation Center for Biomedical Engineering, School of Engineering Sciences, Huazhong University of Science and Technology, Wuhan, Hubei 430074, China

[3]MoE Key Laboratory of Quark and Lepton Physics and College of Physics Science and Technology, Central China Normal University, Wuhan 430079, China

[*]Corresponding authors: p_wang@hust.edu.cn
gpzhang@mail.ccnu.edu.cn

[#]These authors contributed equally

[*]Corresponding authors: p_wang@hust.edu.cn
gpzhang@mail.ccnu.edu.cn

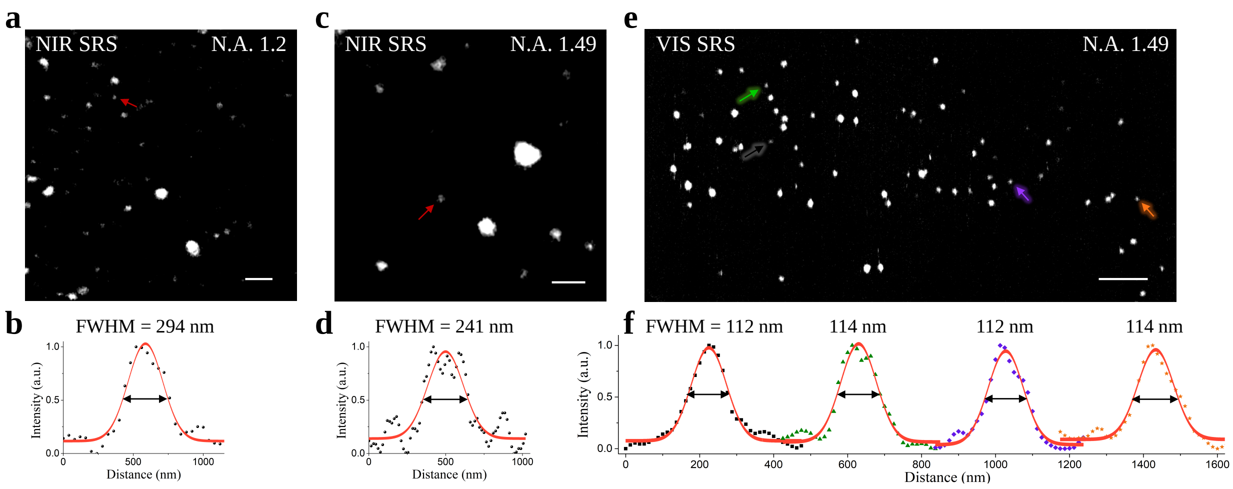

Supplementary Fig. 1 SRS images of PS beads. a, **c**, NIR SRS images. **b**, **d**, Cross section profile of selected beads in **a** and **c**, respectively. **e**, Visible SRS image. **f**, Cross section profile of four selected beads in **e**. The colors in **f** are corresponding to different beads in **e**. Scale bars, 2 μm.

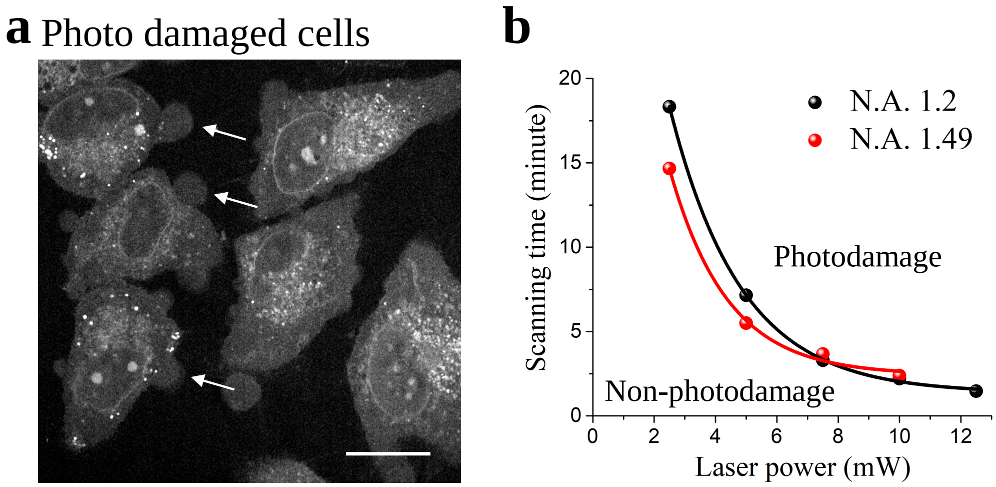

Supplementary Fig. 2 Evaluation of photodamage to living HeLa cells. a, Photo damaged cells indicated by blebbings of the cell membranes (arrows). **b**, Dependence of photodamage on continuous laser scanning time and the total laser power. The ratio of laser power between Stokes and pump beams was set at 1.5. Scale bars, 20 μm.

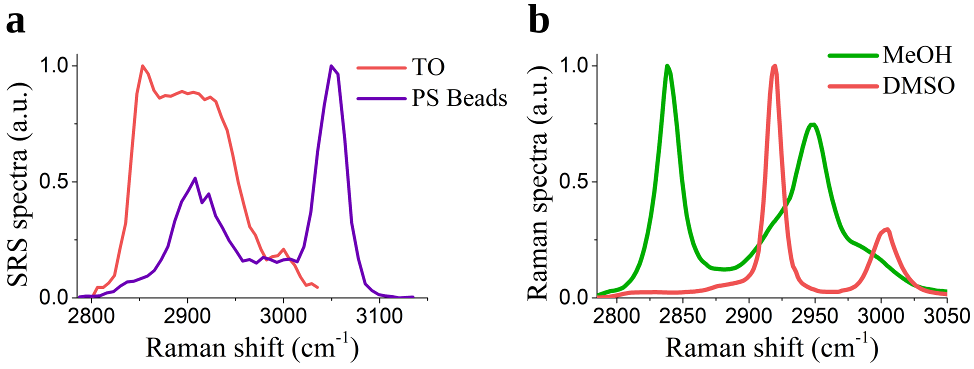

Supplementary Fig. 3 a, Visible SRS spectra. **b**, Spontaneous Raman spectra.

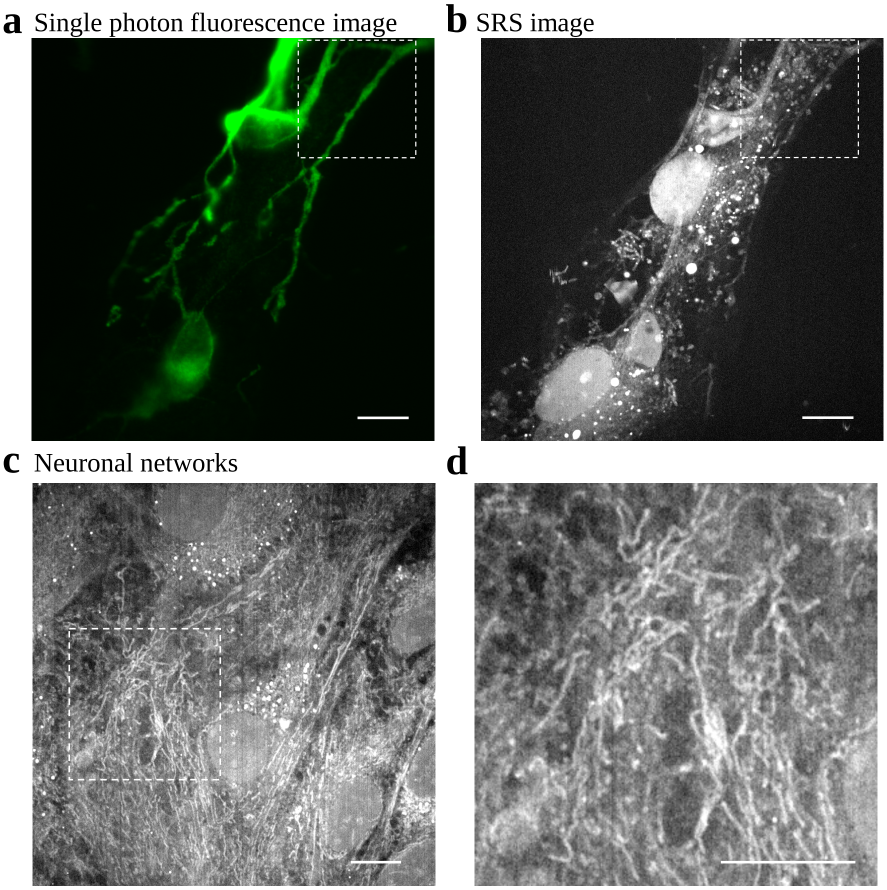

Supplementary Fig. 4 Images of neurons. a, Fluorescence image of Anti-MAP2 immuno-labelled neurons. **b**, Visible SRS image of neurons and glial cells corresponding to **a**. **c**, Visible SRS image of neurons and glial cells closed to glass. **d**, Zoomed-in image of indicated region in **c**. Scale bars, 10 μm.

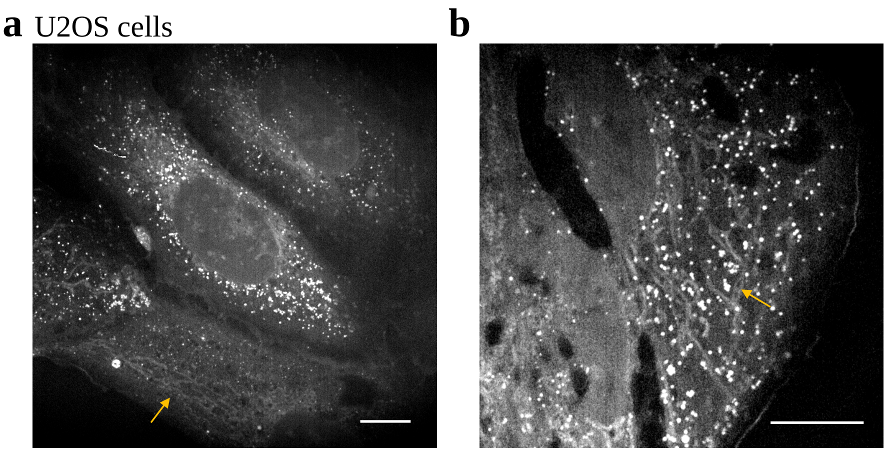

Supplementary Fig. 5 Visible SRS images of U2OS cells. Scale bars, 10 μm.

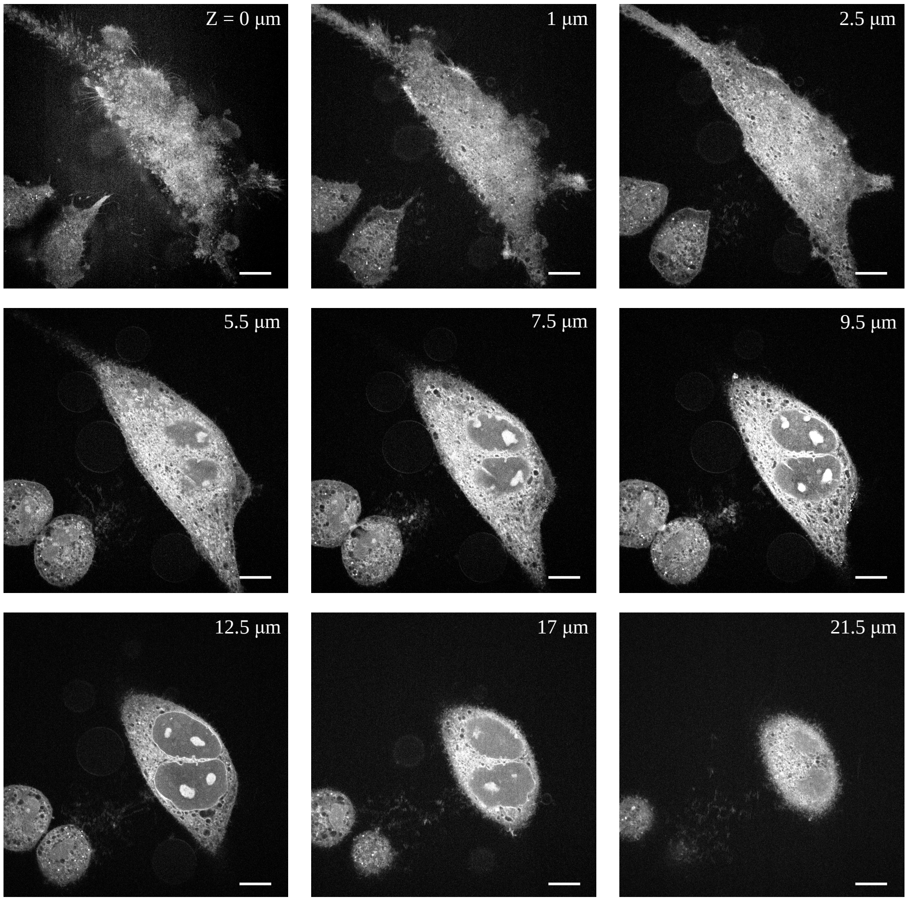

Supplementary Fig. 6 Image stack of HeLa cells at different layers. Scale bars, 10 μm.

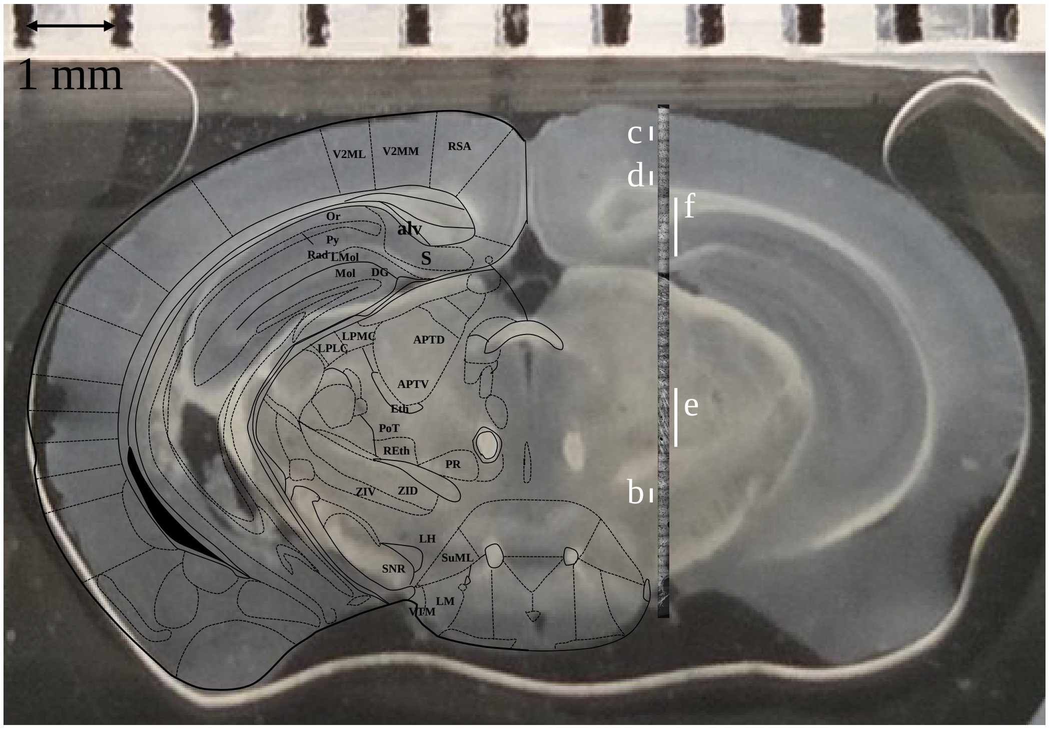

Supplementary Fig. 7 Registration of coronal brain slice and atlas. The inspected area by Visible SRS was indicated. The ruler above the image was 1 mm per interval.

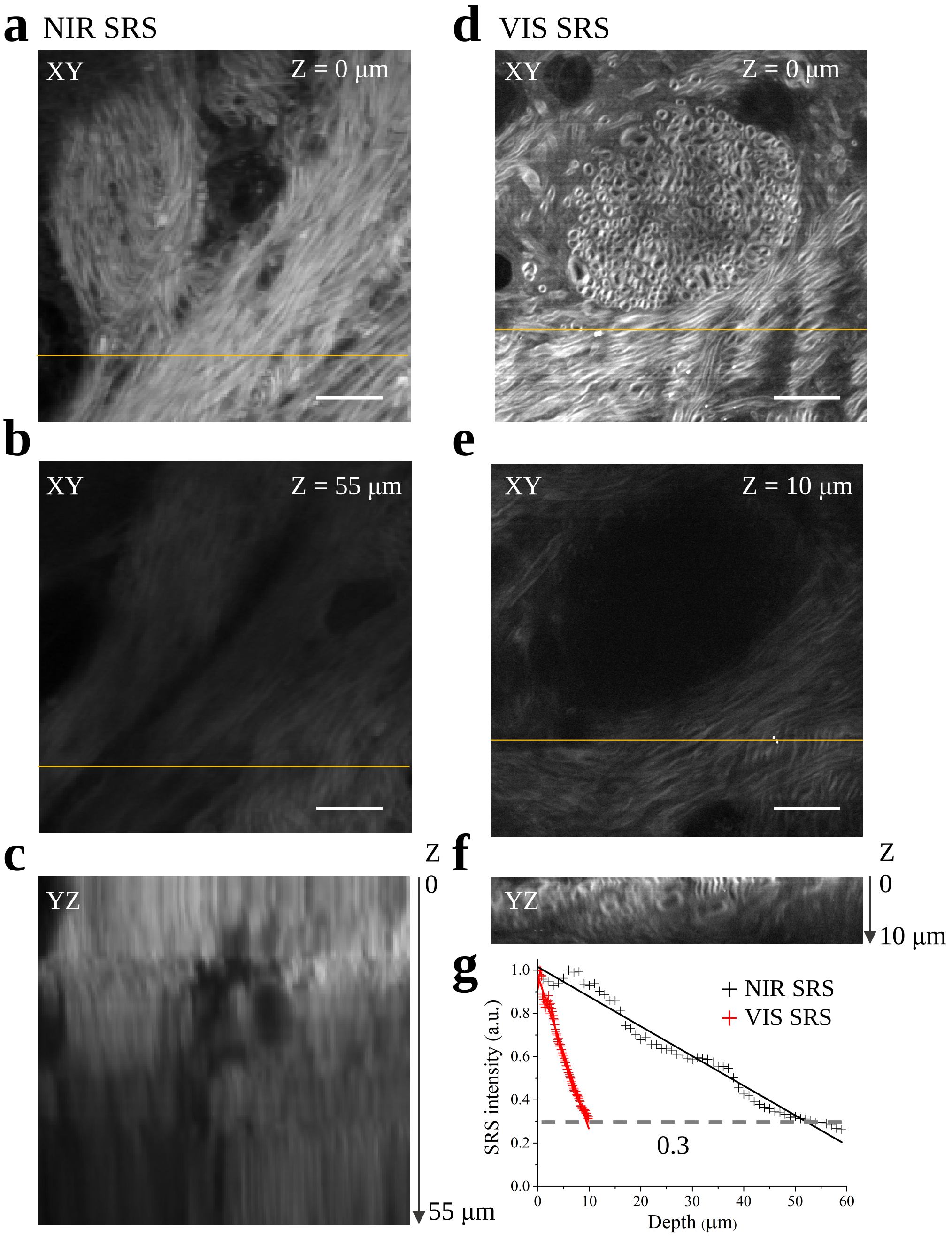

Supplementary Fig. 8 Penetration depth test via 3D imaging of brain tissue. a-b, NIR SRS images of brain tissue slice at the depth of 0 and 55 μm, respectively. **c**, NIR SRS image along YZ plane. The selected plane is indicated by the yellow line in **a** and **b**. **d-e**, Visible SRS images of brain tissue slice at the depth of 0 and 10 μm, respectively. **f**, Visible SRS image along YZ plane. **g**, Dependence of SRS intensity on the penetration depth. Scale bars, 10 μm.

Supplementary Fig. 9 SRS images of a long strip area for mouse brain tissue. Total 56 SRS images were stitched to generate a large scale map. The total inspected area by blue SRS was 120×5,207 μm^2 with 2,000×86,776 pixels. (See separated attachment)

Supplementary Video. Image stack of the HeLa cells. Interval along z axis was 500 nm.
